# Bistable Mathematical Model of Neutrophil Migratory Patterns After LPS-Induced Epigenetic Reprogramming

**DOI:** 10.3389/fgene.2021.633963

**Published:** 2021-02-23

**Authors:** Stanca M. Ciupe, Brittany P. Boribong, Sarah Kadelka, Caroline N. Jones

**Affiliations:** ^1^Department of Mathematics, Virginia Tech, Blacksburg, VA, United States; ^2^Division of Pediatric Pulmonology, Massachusetts General Hospital, Boston, MA, United States; ^3^Department of Environmental Systems Science, ETH Zurich, Zurich, Switzerland; ^4^Department of Bioengineering, University of Texas, Dallas, TX, United States

**Keywords:** neutrophil migration, mathematical model, lipopolysaccharide (LPS), bistability, cellular decision-making

## Abstract

The highly controlled migration of neutrophils toward the site of an infection can be altered when they are trained with lipopolysaccharides (LPS), with high dose LPS enhancing neutrophil migratory pattern toward the bacterial derived source signal and super-low dose LPS inducing either migration toward an intermediary signal or dysregulation and oscillatory movement. Empirical studies that use microfluidic chemotaxis-chip devices with two opposing chemoattractants showed differential neutrophil migration after challenge with different LPS doses. The epigenetic alterations responsible for changes in neutrophil migratory behavior are unknown. We developed two mathematical models that evaluate the mechanistic interactions responsible for neutrophil migratory decision-making when exposed to competing chemoattractants and challenged with LPS. The first model, which considers the interactions between the receptor densities of two competing chemoattractants, their kinases, and LPS, displayed bistability between high and low ratios of primary to intermediary chemoattractant receptor densities. In particular, at equilibrium, we observe equal receptor densities for low LPS (< 15ng/mL); and dominance of receptors for the primary chemoattractant for high LPS (> 15ng/mL). The second model, which included additional interactions with an extracellular signal-regulated kinase in both phosphorylated and non-phosphorylated forms, has an additional dynamic outcome, oscillatory dynamics for both receptors, as seen in the data. In particular, it found equal receptor densities in the absence of oscillation for super-low and high LPS challenge (< 0.4 and 1.1 <LPS< 375 ng/mL); equal receptor densities with oscillatory receptor dynamics for super-low LPS (0.5 < LPS< 1.1ng/mL); and dominance of receptors for the primary chemoattractant for super-high LPS (>376 ng/mL). Predicting the mechanisms and the type of external LPS challenge responsible for neutrophils migration toward pro-inflammatory chemoattractants, migration toward pro-tolerant chemoattractants, or oscillatory movement is necessary knowledge in designing interventions against immune diseases, such as sepsis.

## 1. Introduction

Researchers have recently challenged the dogma that innate immunity is the same at every challenge. It has been shown that macrophages are able to develop different kinds of memory depending on the type of priming they encounter via epigenetic reprogramming (Yuan et al., 2016a,b). For instance, they can develop a memory phenotype that leads them to be less reactive or even tolerant to a challenge, or they can develop a memory phenotype that leads them to have an enhanced response to a challenge. This same concept has recently been shown by us for neutrophil migratory decision-making, and it is thought that the response is influencing the outcomes of infectious diseases. For example, in sepsis or COVID-19 infection, the immune system overreacts because of underlying low-grade inflammation that primes neutrophils into choosing between tolerant and inflammatory migratory phenotypes (Alves-Filho et al., 2005, 2010). As a result, neutrophils can migrate to healthy organs and unleash their anti-microbial arsenal in healthy tissue, leading to organ failure in the lungs, kidney, or heart. The mechanisms underlying trained innate immunity have not been fully elucidated, with epigenetic modifications playing a key role in the induction of *innate memory or training* (Pillay et al., 2010; Demaret et al., 2015). In this study we investigate innate memory in the context of neutropil migratory decision-making.

The ability of neutrophils to migrate plays a pivotal role in a cell's ability to clear infections and resolve inflammation. During infection and inflammation, chemoattractants are released, signaling and activating neutrophils in the bloodstream. Neutrophils must be able to precisely migrate within the tissue to the specific site of infection, without being diverted toward other locations, in a process called chemotaxis. Chemotaxis is a highly regulated process that involves activation of various pathways and downstream polarization of the cell (Kolaczkowska and Kubes, 2013). The first step in chemotaxis is recognition of chemoattractants by the cell. Cells have specific receptors on their surface for various chemoattractants. These chemoattractant receptors are G protein-coupled receptors (GPCRs), which are regulated by a variety of G protein-coupled receptor kinases (GRKs) (Murphy, 1994; Dianqing, 2005). When bound by a specific agonist, in this case a chemoattractant, the GPCRs undergo phosphorylation, which unbinds the G proteins and desensitizes the receptor. This leads to internalization of the receptor, activation of downstream signaling pathways, and activation of cellular responses, such as cell polarization and chemotaxis (Murphy, 1994; Dianqing, 2005; Futosi et al., 2013). After internalization, receptors can be recycled back to the cell surface, where they can again be bound by the receptor's agonist. This process is crucial in chemotaxis, as it allows the cell to continue sensing the chemoattractant and migrate in its direction (Neel et al., 2005). Most chemoattractant receptors are similar in their response to ligand-binding; however there are slight differences in the activated signaling pathways (Heit et al., 2002, 2008). Within the tissue, neutrophils are exposed to several chemoattractants at once, originating from pathogens, cells within the tissue, the endothelium, and several other sources (Kolaczkowska and Kubes, 2013). Cells must prioritize these signals to properly clear the pathogen. It has been hypothesized that neutrophils have an internal hierarchy, where chemoattractants derived from bacterial sources and the complement system, such as fMLP and C5a (Heit et al., 2002; Petri and Sanz, 2018), take precedent over intermediary chemoattractants, such as LTB_4_ and IL-8, which are secreted by other immune cells. This leads to neutrophils migrating toward end-target chemoattractants over intermediary chemottractants in a competitive environment (Heit et al., 2002, 2008; Wang et al., 2016b), allowing neutrophils to prioritize an invading pathogen. This hierarchy is thought to occur through the activation of differing signaling pathways, where end-target chemoattractants signal through p38 MAPK and intermediary chemoattractants signal through PI3K (Heit et al., 2002, 2008).

The highly controlled migration of neutrophils toward the site of an infection, as well as their dynamic interaction with pathogens, can be altered when they are pre-conditioned with Lipopolysaccharides (LPS) to induce endotoxin priming. In previous work, we showed that training with high dose LPS (100 ng/mL) enhances neutrophil migration toward the end-target, bacterial derived, source signal fMLP. By contrast, training with super-low dose LPS (1 ng/mL) alters neutrophil migratory phenotypes, which either migrate toward the intermediary signal LTB_4_ or become dysregulated and exhibit oscillatory migratory patterns (Jones et al., 2016; Boribong et al., 2019). While the empirical data shows that neutrophils trained with LPS change migratory phenotype, it does not give information on the molecular mechanisms responsible for the difference in behavior. The migratory decision-making process is finely governed by complex signaling networks that dynamically receive and interpret molecular and cellular signals from outside and within. The intrinsic complexity of immune cell decision-making processes has created difficulty for experimental immunologists to determine the mechanisms of disease, in spite of expansive experimental studies with conventional reductionist cellular and molecular approaches. It is increasingly recognized that cross-disciplinary studies combining experimental and mathematical modeling approaches are critically required.

**Figure 1:**
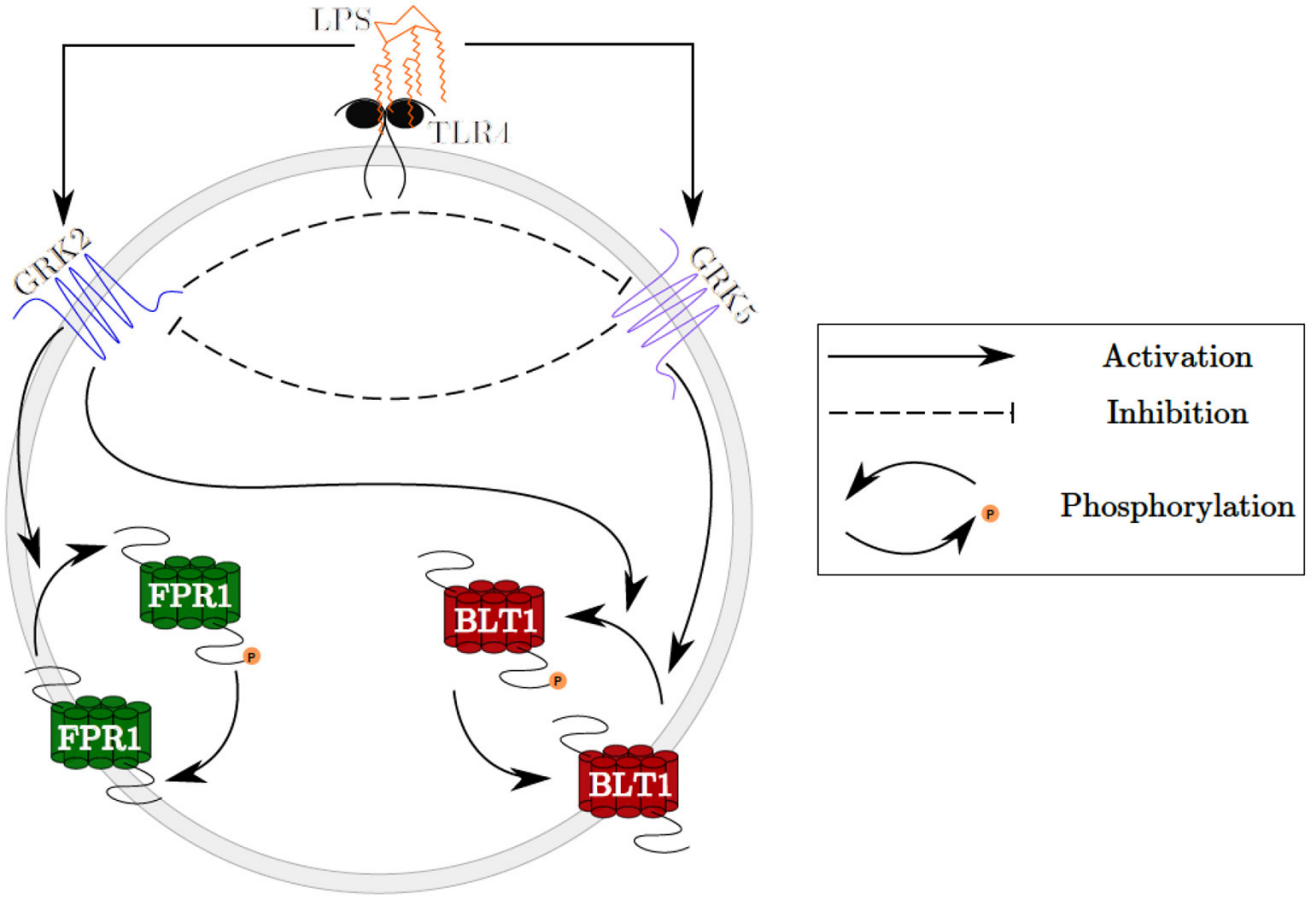
Schematic representation of the GRK2 and GRK5 mutual inhibition.

In this study, we investigate the molecular mechanisms of neutrophil migratory decision-making in the presence of competing chemoattractants and external challenge with LPS, by building deterministic mathematical models of interaction between two chemoattractant receptors, Formyl Peptide Receptor 1 (FPR1) and Leukotriene B4 Receptor 1 (BLT1), and key molecules involved in their regulation. We are interested in determining the relationship between the receptor dynamics and migration pattern, and in quantifying the LPS dose resulting in neutrophils migration toward a pro-inflammatory chemoattractant, toward a pro-resolution chemoattractant, or in neutrophils dysregulation and oscillation (Fan and Malik, 2003; Liu et al., 2012; Byrne et al., 2014). The model will qualitatively match the experimental results of our previous work, where stimulation with a super-low concentration of LPS will result in greater BLT1 over FPR1, and stimulation with a high concentration of LPS will result in greater FPR1 over BLT1 (Boribong et al., 2019). We construct a model with bistable behavior, with the motif for bistability coming from the non-linear mutual inhibition of GRK2 and GRK5 (see Figure 1). The dual inhibition leads to the activation of different signaling pathways (p38/JNK vs. ERK), leading to differences in functional neutrophil migration (Davenport et al., 2020). Both GRK2 and GRK5 have been demonstrated to be critical mediators of the molecular alterations that occur in the inflammatory disorders, but the complex mutual inhibition interaction has largely been ignored (Philipp et al., 2014). Mathematical models have been used before to model cellular decision-making (Day et al., 2006; Kadelka et al., 2019), neutrophil chemotaxis (Ionides et al., 2004; Postma and van Haastert, 2016; Bayani et al., 2020), immune responses (Reynolds et al., 2006; Fischer, 2008; Nelson et al., 2009; Vodovotz et al., 2009) and bistable dynamics (Ciupe et al., 2007, 2018; Leber et al., 2016).

## 2. Methods

### 2.1. Mathematical Model of Migratory Decision-Making

**Figure 2:**
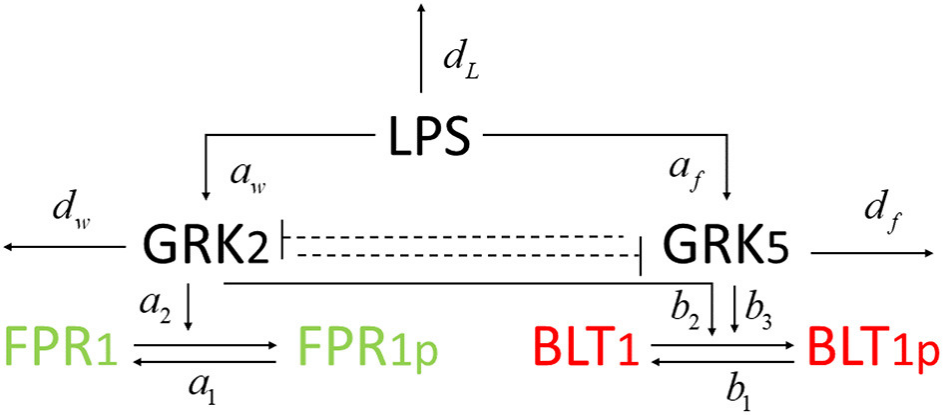
Network diagram for model (1).

We developed a novel system of differential equations based on diagram in Figure 2, which describes the interactions between [LPS], kinases [GRK2] and [GRK5], the receptor for end-target chemoattractant fMLP, [*FPR*1], and the receptor for intermediary chemoattractant LTB_4_, [BLT1]. Priming by LPS occurs through activation of both GRK2 and GRK5 (Prossnitz et al., 1995; Arraes et al., 2006; Sorriento et al., 2008; Wang et al., 2016a). For simplicity, we model linear effects of LPS on the kinases' activity. In particular, we assume that the GRK2 activation occurs at rate *c*_*w*_+*a*_*w*_[*LPS*], with *c*_*w*_ and *a*_*w*_ being the LPS-independent and LPS-dependent activation rates. Similarly, GRK5 activation occurs at rate *c*_*f*_+*a*_*f*_[*LPS*], with *c*_*f*_ and *a*_*f*_ being the LPS-independent and LPS-dependent activation rates. The two kinases mutually inhibit one another. We model inhibition of GRK2 via GRK5 at rate $1/\left(b_{fw}+{\left[GRK5\right]}^{n}\right)$ and inhibition of GRK5 via GRK2 at rate 1/(*b*_*wf*_+[*GRK*2]), where *b*_*fw*_ and *b*_*wf*_ are the mutual inhibition rates of GRK2 by GRK5 and GRK5 by GRK1, respectively. *n* is the cooperativity coefficient. We assumed increased cooperativity in GRK2 inhibition by GRK5, but not the inhibition of GRK5 by GRK2. The results are preserved if the same cooperativity is included in the GRK5 inhibition by GRK2 (not shown). We assume GRK2 and GRK5 decay at per capita rates *d*_*w*_ and *d*_*f*_, respectively, with GRK5 decay being modeled in a density dependent manner, with the GRK5 value where the decay is half-maximal being given by parameter *b*_*f*_.

The chemoattractant receptors FPR1 and BLT1 internalize from the plasma membrane into the cell via phosphorylation (Magalhaes et al., 2012; Mócsai et al., 2015). We assume that the number of receptors on a cell is conserved and, through the process of dephosphorylation, the receptors are recycled and brought back to the surface of the cell. Thus, we have conservation laws of the total number of the receptor equalling the sum of the non-phosphrylated and phosphorylated receptor, [*FPR*1]_*total*_ = [*FPR*1]+[*FPR*1_*p*_] and [*BLT*1]_*total*_ = [*BLT*1]+[*BLT*1_*p*_]. The process of receptor phosphorylation and dephosphorylation is modeled using Hill-type functions. In particular, FPR1 is produced through dephosphorylation, modeled by a Michaelis-Menten term *a*_1_([*FPR*1]_*total*_ − [*FPR*1])/(*J*_*F*1_+[*FPR*1]_*total*_ − [*FPR*1]), where *a*_1_ is maximal production and *J*_*F*_1__ is the receptor quantity where dephosphorylation is half-maximal. Similarly, FPR1 is lost through phosphorylation, which is enhanced in the presence of GRK2 (Wang et al., 2016a). We model this by a Hill-type function *a*_2_[*FPR*1][*GRK*2]/(*J*_*F*2_+[*FPR*1]), where *a*_2_ is the maximal rate and *J*_*F*_2__ is the receptor quantity where phosphorylation is half-maximal.

BLT1 is produced through dephosphorylation, modeled by a Michaelis-Menten term *b*_1_([*BLT*1]_*total*_ − [*BLT*1])/(*J*_*B*1_+[*BLT*1]_*total*_ − [*BLT*1]), where *b*_1_ is the maximal production rate and *J*_*B*_1__ is the receptor quantity where dephosphorylation is half-maximal. BLT1 is lost through phosphorylation, which is enhanced in the presence of both GRK2 and GRK5 (Gaudreau et al., 2002; Chen et al., 2004). We model this by a Hill-type function [*BLT*1](*b*_2_[*GRK*2]+*b*3[*GRK*5])/(*J*_*B*2_+[*BLT*1]), where *b*_2_ are *b*_3_ are maximal decay rates and *J*_*B*_2__ is the receptor quantity where phosphorylation is half-maximal. We assume a single LPS dose, after which LPS decays exponentially at a rate *d*_*L*_ (Kadelka et al., 2019). The dynamical system describing these interactions is given by:

(1) $$\begin{array}{l} \begin{array}{c} \frac{d\left[GRK2\right]}{dt}=\frac{c_{w}+a_{w}\left[LPS\right]}{b_{fw}+{\left[GRK5\right]}^{n}}-d_{w}\left[GRK2\right],\\ \frac{d\left[GRK5\right]}{dt}=\frac{c_{f}+a_{f}\left[LPS\right]}{b_{wf}+\left[GRK2\right]}-d_{f}\frac{\left[GRK5\right]}{b_{f}+\left[GRK5\right]},\\ \frac{d\left[FPR1\right]}{dt}=\frac{a_{1}\left({\left[FPR1\right]}_{total}-\left[FPR1\right]\right)}{J_{F1}+{\left[FPR1\right]}_{total}-\left[FPR1\right]}\\ \;-a_{2}\left[GRK2\right]\frac{\left[FPR1\right]}{J_{F2}+\left[FPR1\right]},\\ \frac{d\left[BLT1\right]}{dt}=\frac{b_{1}\left({\left[BLT1\right]}_{total}-\left[BLT1\right]\right)}{J_{B1}+{\left[BLT1\right]}_{total}-\left[BLT1\right]}-(b_{2}\left[GRK2\right]\\ \;+b3\left[GRK5\right])\frac{\left[BLT1\right]}{J_{B2}+\left[BLT1\right]},\\ \;\frac{d\left[LPS\right]}{dt}=-d_{L}\left[LPS\right]. \end{array} \end{array}$$

We are interested in determining the ratio between the cells that migrate toward the primary and those that migrate toward the intermediary chemoattractants given, as a proxy, by the ratio of their receptors FPR1/BLT1, when initial LPS is varied.

#### 2.1.1. Experimental Data

**Figure 3:**
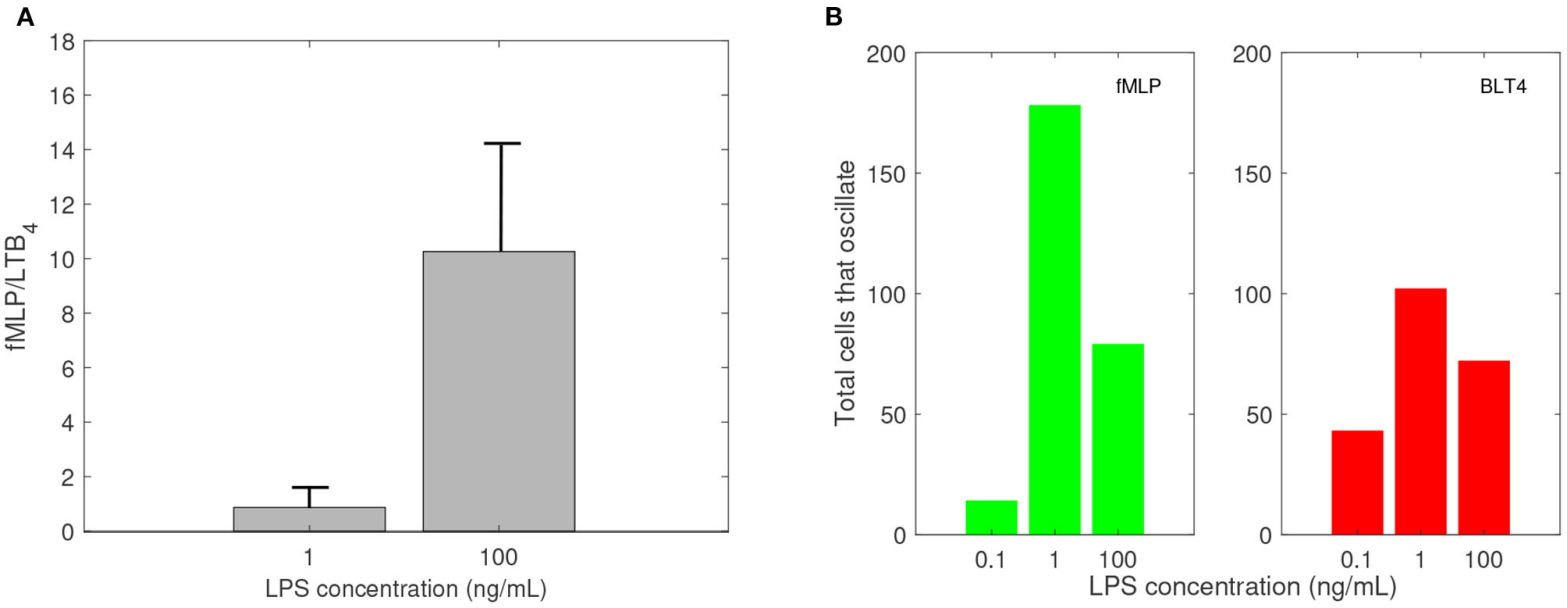
Empirical data: **(A)** Ratio of fMLP/LTB4 cell migration, and **(B)** number of cells that oscillate (change direction at least three times) while migrating toward fMLP (green) and LTB4 (red) vs. LPS concentration in ng/mL. Data reproduced from Boribong et al. (2019).

In previous research, we used a microfluidic competitive chemotaxis-chip device to measure the migratory decision-making process of dHL-60 cells, a model neutrophil cell line, 5 h after they were pre-challenged with super-low-dose (1 ng/mL) and high-dose (100 ng/mL) of LPS in the presence of two competing chemoattractants, LTB4 and fMLP (Boribong et al., 2019). Challenging the cells with a super-low dose of LPS resulted in fMLP/LTB4 ratio of 0.8672. Challenging the cells with a high dose of LPS (100 ng/mL) resulted in fMLP/LTB4 ratio of 10.2646 (see Figure 3A).

#### 2.1.2. Parameter Values

There are approximately 40, 000 FPR1 and 13, 333 BLT1 receptors on each neutrophil (Schneider et al., 2012). We therefore set initial conditions to [*FPR*1](0) = 40, 000 and [*BLT*1](0) = 13, 333. The reported GRK2/GRK5 ratio is 1.5 (Arraes et al., 2006). We choose initial conditions [*GRK*2](0) = 0.75 and [*GRK*5](0) = 0.5, to preserve this ratio. The reported GRK2 half-life varies between 60 min in HEK, COS-7, Jurkat, C6 glioma cells (Penela et al., 1998) and 20–24 h in undifferentiated HL-60 cells (Luo and Benovic, 2003). We choose a shorter half-life of 1 h, which corresponds to the GRK decay rate *d*_*w*_ = log(2)/1 = 0.69 per hour. The reported GRK5 life-span is 3 h (Wu et al., 2012), which corresponds to the GRK5 decay rate *d*_*f*_ = 1/3 = 0.33 per hour. The FPR1's phosphorylation half-life is 15 s (Leoni et al., 2015). We choose both the phosphorylation and dephosphorylation rates based on this value, *a*_1_ = *a*_2_ = log(2) × 3, 600/15 = 166 per hour. BLT1 phosphorylation's half-life is 120 s (Gaudreault et al., 2005). We choose both the phosphorylation and dephosphorylation rates based on this value, *b*_1_ = *b*_2_ = *b*_3_ = log(2) × 3, 600/120 = 20 per hour. As in our previous work (Kadelka et al., 2019), the LPS degradation rate is *d*_*L*_ = 0.1 per day. For simplicity, we fix most unknown parameters at one, *a*_*w*_ = *c*_*f*_ = *a*_*f*_ = *b*_*f*_ = *J*_*F*1_ = *J*_*f*2_ = *J*_*B*1_ = *J*_*B*2_ = 1. Moreover, *c*_*w*_ = 15, $b_{fw}=5\times 10^{-4}$, *b*_*wf*_ = 0.13 and *n* = 3. The parameter values are summarized in Table 1.

**Table 1:** Parameters and initial conditions used in model 1.

**Parameter**	**Description**	**Value**	**References**
*c*_*w*_	[*LPS*]-independent [*GRK*2] activation	15	
*a*_*w*_	[*LPS*]-dependent [*GRK*2] activation	1	
*d*_*w*_	[*GRK*2] degradation	0.69	Penela et al., 1998; Luo and Benovic, 2003
*b*_*fw*_	[*GRK*2] inhibition by [*GRK*5]	5 × 10^−4^	
*c*_*f*_	[*LPS*]-independent [*GRK*5] activation	1	
*a*_*f*_	[*LPS*]-dependent [*GRK*5] activation	1	
*d*_*f*_	[*GRK*5] degradation	0.33	Wu et al., 2012
*b*_*wf*_	[*GRK*5] inhibition by [*GRK*2]	0.13	
*b*_*f*_	[*GRK*5] where degradation is half-maximal	1	
*n*	Hill coefficient	3	
*a*_*i*_, *i* = {1, 2}	phosphorylation and dephosphorylation rates	166	Leoni et al., 2015
*b*_*i*_ *i* = {1, 2, 3}	phosphorylation and dephosphorylation rates	20	
*J*_*i*_, *i* = {*F*1, *F*2, *B*1, *B*2}	values where phosphorylation is half-maximal	1	Gaudreault et al., 2005
[*FRP*1]_*total*_	Total [*FRP*1] receptors	40, 000	Schneider et al., 2012
[*BLT*1]_*total*_	Total [*BLT*1] receptors	13, 333	Schneider et al., 2012
*d*_*L*_	[*LPS*] loss	0.1	Kadelka et al., 2019
**Initial conditions**	**Description**	**Value**	**References**
[*GRK*2](0)	Initial [*GRK*2] value	0.75	Arraes et al., 2006
[*GRK*5](0)	Initial [*GRK*5] value	0.5	Arraes et al., 2006
[*FPR*1](0)	Initial [*FPR*1] value	40,000	Schneider et al., 2012
[*BLT*1](0)	Initial [*BLT*1] value	13,333	Schneider et al., 2012
[*LPS*](0)	Initial [*LPS*] value	Varied	Boribong et al., 2019

### 2.2. Mathematical Model of Oscillatory Movement

We coupled system (1) with an oscillator describing the dynamics of non-phosphorylated and phosphorylated extracellular signal-regulated kinases (ERK), [*ERK*] and [*ERK*_*p*_], that are participating in an autocatalytic reaction with the help of intermediate non-phosphorylated and phosphorylated enzymes, [*E*] and [*E*_*p*_] (see Figure 7). We assume that [*ERK*] activation is LPS-dependent and occurs at rate *k*_1_[*LPS*]. The phosphorylated [*ERK*_*p*_] decays at rate *k*_2_. The phosphorylated enzyme [*E*_*p*_] follows the following reaction:

(2) $$\begin{array}{l} \frac{d\left[E_{p}\right]}{dt}=k_{E1}\left[ERK_{p}\right]\frac{\left[E\right]}{J_{E_{1}}+\left[E\right]}-\frac{k_{E2}\left[E_{p}\right]}{J_{E_{2}}+\left[E_{p}\right]}, \end{array}$$

where *k*_*Ei*_ are the dephosphorylation and phosphorilation rates and *J*_*E*_*i*__ are the phosphorylation and dephosphorylation half-maximal rates, *i* = {1, 2}. If we assume chemical equilibrium, [*E*_*p*_]+[*E*] = 1, and *k*_3_ = *k*_*E*2_/*k*_*E*1_, we obtain that:

(3) $$\begin{array}{l} \left[E_{p}\right]=\frac{X-\sqrt{X^{2}-4\left(k_{3}-\left[ERK_{p}\right]\right)\left[ERK_{p}\right]J_{E2}}}{2\left(k_{3}-\left[ERK_{p}\right]\right)}\\ \;=\frac{2J_{E2}\left[ERK_{p}\right]}{X+\sqrt{X^{2}-4\left(k_{3}-\left[ERK_{p}\right]\right)\left[ERK_{p}\right]J_{E2}}}\\ \;=\text{G}\left(\left[ERK_{p}\right],k_{3},J_{E1},J_{E2}\right) \end{array}$$

where *X* = *k*_3_ − [*ERK*_*p*_] + *k*_3_*J*_*E*1_ + *J*_*E*2_[*ERK*_*p*_] and G([*ERK*_*p*_], *k*_3_, *J*_*E*1_, *J*_*E*2_)) is the Goldbeter-Koshland function (Goldbeter and Koshland, 1981). Hence, the phosphorylation of [*ERK*] occurs at rate (*k*_0*s*_ + *k*_0_G([*ERK*_*p*_], *k*_3_, *J*_*E*1_, *J*_*E*2_))[*ERK*]. Lastly, the LPS is constant at all times LPS=[*LPS*](0), to account for positive long-term [*ERK*] levels. The model is given by the system:

(4) $$\begin{array}{l} \begin{array}{c} \;\frac{d\left[ERK\right]}{dt}=k_{1}\left[LPS\right]-\left(k_{0s}+k_{0}\text{G}\left(\left[ERK_{p}\right],k_{3},J_{E1},J_{E2}\right)\right)\left[ERK\right],\\ \frac{d\left[ERK_{p}\right]}{dt}=\left(k_{0s}+k_{0}\text{G}\left(\left[ERK_{p}\right],k_{3},J_{E1},J_{E2}\right)\right)\left[ERK\right]-k_{2}\left[ERK_{p}\right],\\ \frac{d\left[GRK2\right]}{dt}=\frac{c_{w}+a_{w}\left[ERK_{p}\right]}{b_{fw}+{\left[GRK5\right]}^{n}}-d_{w}\left[GRK2\right],\\ \frac{d\left[GRK5\right]}{dt}=\frac{c_{f}+a_{f}\left[ERK_{p}\right]}{b_{wf}+\left[GRK2\right]}-d_{f}\frac{\left[GRK5\right]}{b_{f}+\left[GRK5\right]},\\ \frac{d\left[FPR1\right]}{dt}=\frac{a_{1}\left({\left[FPR1\right]}_{total}-\left[FPR1\right]\right)}{J_{F1}+{\left[FPR1\right]}_{total}-\left[FPR1\right]}\\ \;-a_{2}\left[GRK2\right]\frac{\left[FPR1\right]}{J_{F2}+\left[FPR1\right]},\\ \frac{d\left[BLT1\right]}{dt}=\frac{b_{1}\left({\left[BLT1\right]}_{total}-\left[BLT1\right]\right)}{J_{B1}+{\left[BLT1\right]}_{total}-\left[BLT1\right]}-(b_{2}\left[GRK2\right]\\ \;+b3\left[GRK5\right])\frac{\left[BLT1\right]}{J_{B2}+\left[BLT1\right]}. \end{array} \end{array}$$

#### 2.2.1. Experimental Data

Experimental results reported that neutrophils treated overnight with LPS may lose their ability to move up the chemoattractant gradient, become disoriented, and display oscillatory behavior (Boribong et al., 2019). Moreover, the highest number of cells to display such oscillatory behavior occurs following LPS exposure with super-low dose (1 ng/mL) (see Figure 3B) (Boribong et al., 2019).

#### 2.2.2. Parameter Values

We assume that initially [*ERK*](0) = 5 and [*ERK*_*p*_](0) = 0.1. Kinase [*ERK*] is produced at rate *k*_1_ = 0.3 and phosphorylated at rate *k*_0*s*_ = 0.01. Kinase [*ERKp*] is lost at rate *k*_2_ = 1. Enzyme [E] is phosphorylated, in the presence of [ERK], at rate *k*_0_ = 0.4 and dephosphorylated at rate *k*_3_ = 0.3. The processes are modeled using Michaelis-Menten terms, with densities where phosphorylation/ dephosphorylation are half-maximal being set to *J*_*E*1_ = *J*_*E*2_ = 0.005. All other parameters and initial conditions are as in model (1). The new parameter values are summarized in Table 2.

**Table 2:** Parameters and initial conditions used in model 2.

*k*_0_	[*ERK*]-dependent [*E*] phosphorylation	0.4	
*k*_1_	[*LPS*]-dependent [*ERK*] activation	0.3	
*k*_2_	[*ERKp*] degradation rate	1	
*k*_3_	[*ERK*]-independent [*E*] dephosphorylation	0.3	
*k*_0*s*_	[*ERK*] phosphorylation rate	0.01	
*J*_*i*_, *i* = {*E*1, *E*2}	values where phosphorylation is half-maximal	0.005	
**Initial conditions**	**Description**	**Value**	**References**
[*ERK*](0)	Initial [*ERK*] value	5	
[*ERK*_*p*_](0)	Initial [*ERKp*] value	0.1	

## 3. Results

### 3.1. Bistable FPR1 and BLT1 Dynamics

We evaluated neutrophil migration between end-target chemoattractant fMLP and intermediary chemoattractant LTB_4_ by developing model (1), which considers the interaction between the chemoattractants' receptors, [*FPR*1] and [*BLT*1], the receptors' kinases, [*GRK*2] and [*GRK*5], and [*LPS*]. We quantified the [*FPR*1]/[*BLT*1] ratio for different [*LPS*] doses under the dynamics of system (1), and parameters/initial conditions given in Table 1. Since the experimental data has collected ratios of cell migration 5 h after LPS challenge, we first quantified [*FPR*1]/[*BLT*1] at time *t* = 5.

**Figure 4:**
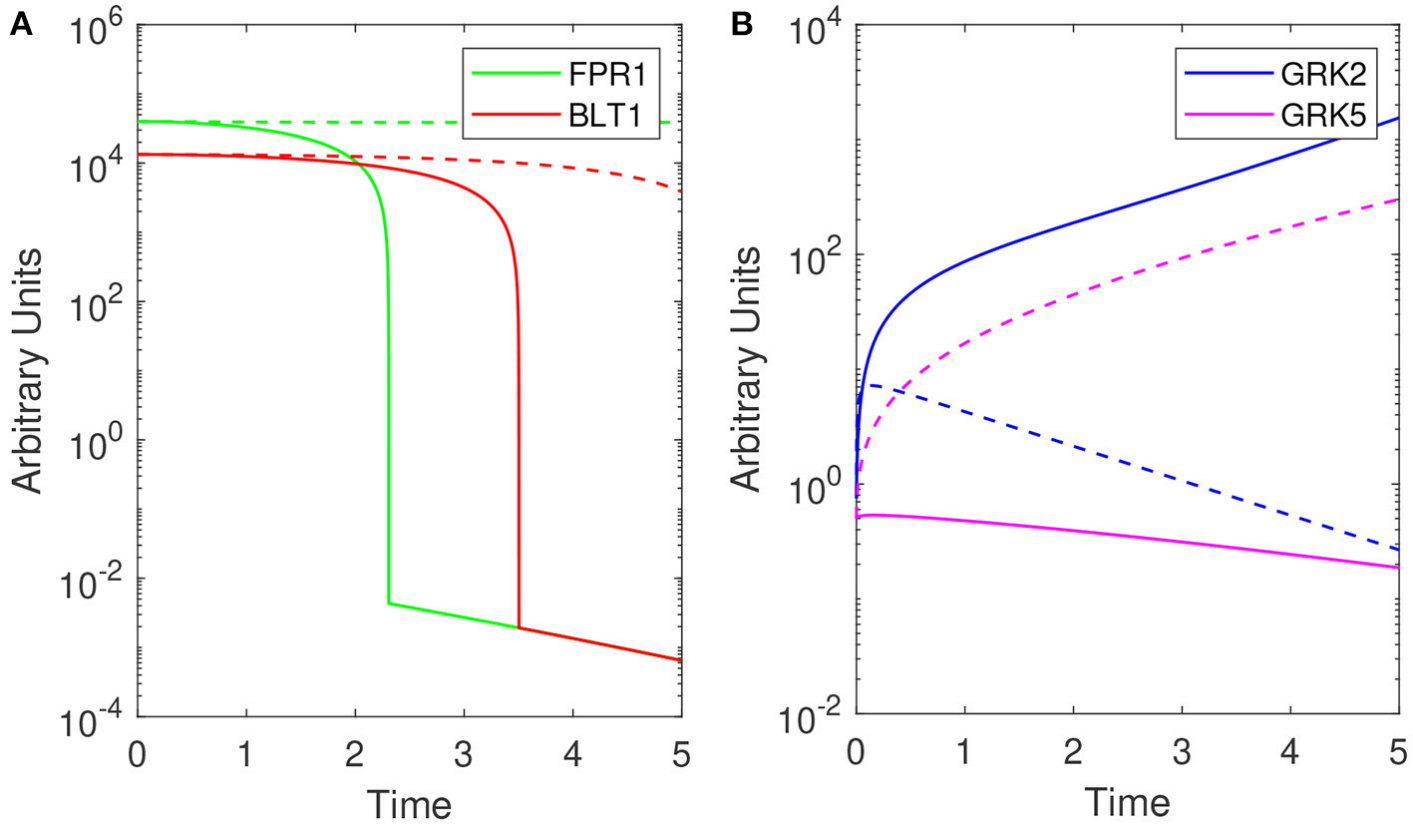
Theoretical results: Dynamics of **(A)** [*FPR*1] (green) and [*BLT*1] (red) and, **(B)** [*GRK*2] (purple) and [*GRK*5] (pink) for [*LPS*](0) = 1 ng/mL (solid lines) and [*LPS*](0) = 100 ng/mL (dashed lines) as given by model (1). Parameters and initial conditions are given in Table 1.

Model (1) exhibits bistable behavior between high and low [*GRK*2] concentrations (low and high [*GRK*5] concentrations), with low [*LPS*] priming leading to high [*GRK*2] production and high [*LPS*] priming leading to low [*GRK*2] production (see Figure 4B). Five hours following challenge with super-low-dose (1 ng/mL) LPS, model (1) predicts the presence of a small number of receptors, which are distributed equally among FPR1 and BLT1, [*FPR*1]/[*BLT*1](5) = 1 (see Figure 4A, solid lines). Under our abstraction this means that, following challenge with 1 ng/mL LPS, an equal number of neutrophils migrated toward the fMLP and LTB4 gradients. Conversely, 5 h following high-dose challenge (100 ng/mL) LPS, model (1) predicts the presence of a large number of receptors of both types, with [*FPR*1] exceeding [BLT1] by one fold, *i.e*, [*FPR*1]/[*BLT*1](5) = 10 (see Figure 4A, solid lines). Under our abstraction this means that a large number of neutrophils have migrated in both directions, with ten times more neutrophils migrating toward fMLP than LTB4. These [*FPR*1]/[*BLT*1] ratios are similar to the fMLP/LTB4 ratios observed in the experimental data (Boribong et al., 2019) (see Figure 3A). We further quantified the [*FPR*1]/[*BLT*1] ratio past the 5 h in the experiment. For the [*LPS*](0) = 1 ng/mL LPS challenge, [*FPR*1]/[*BLT*1](*t*) = 1 for all *t* ≥ 5. By contrast, for the [*LPS*](0) = 100 ng/mL LPS challenge, the [*FPR*1]/[*BLT*1](*t*) ratio becomes larger and larger as *t* increases, with the majority of cells favoring the primary fMLP gradient (not shown).

**Figure 5:**
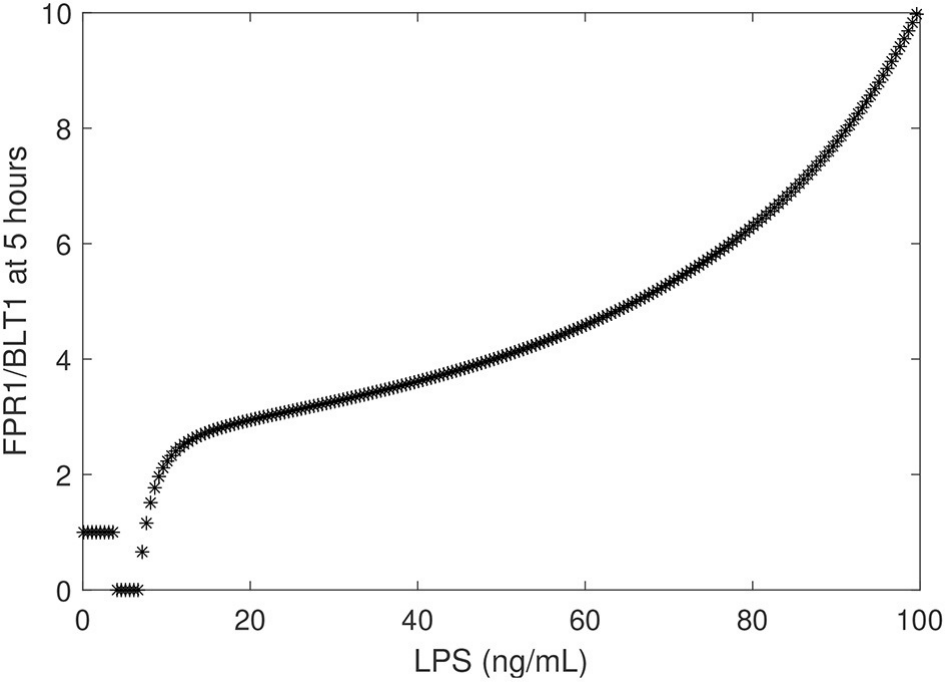
FPR1/BLT1 at time *t* = 5 h, as given by model (1) vs. initial LPS dose.

To determine the relationship between the LPS challenge dose and the FPR1/BLT1 ratio, we derived a graph that quantifies [*FPR*1]/[*BLT*1](5), 5 h following cell priming, as a function of the [*LPS*] dose, predicted by model (1) and parameter values/initial conditions in Table 1. We found that the experimental observation for the super-low-dose (1 ng/mL) LPS, [*FPR*1]/[*BLT*1](5) = 1, is preserved for all challenges with LPS values lower than 3.9 ng/mL. For 4 − 6.7 ng/mL LPS challenge, [*BLT*1] exceeds [*FPR*1] at *t* = 5 h, but the two receptors will eventually reach identical levels at equilibrium. Lastly, the [*FPR*1]/[*BLT*1](5) ratio grows larger than one and keeps increasing for LPS dose >6.7 ng/mL, eventually reaching the experimental prediction of ten, [*FPR*1]/[*BLT*1](5) = 10, for high-dose LPS challenge (100 ng/mL) (see Figure 5) and increasing further as time passes or for higher challenge (not shown).

### 3.2. Long-Term Results and Motifs of Bistability

**Figure 6:**
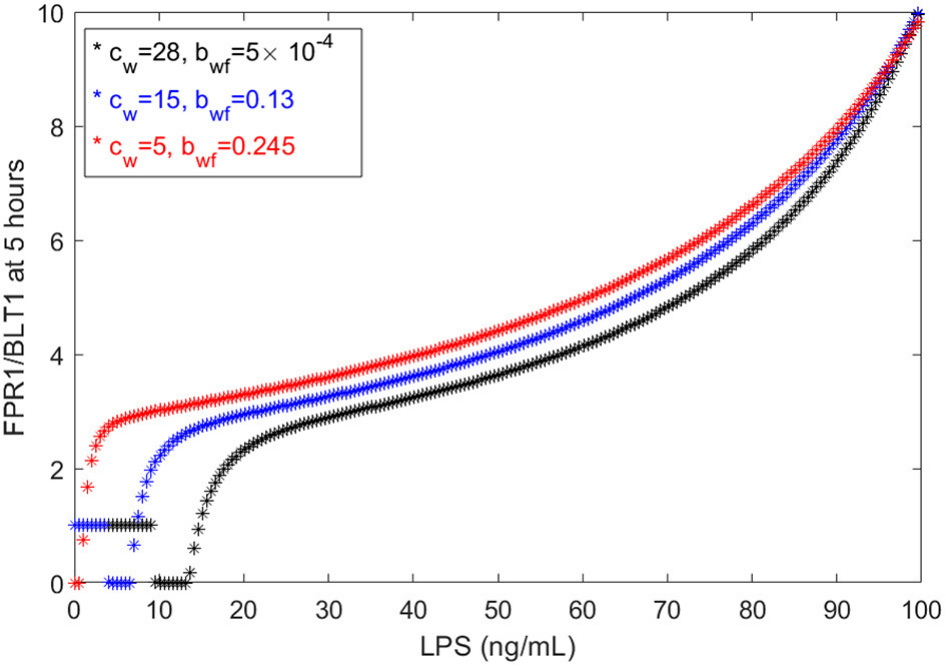
FPR1/BLT1 at time *t* = 5 h, as given by model (1), for *c*_*w*_ = 28, $b_{wf}=5\times 10^{-4}$ (black stars); *c*_*w*_ = 15, *b*_*wf*_ = 0.13 (blue stars); and *c*_*w*_ = 5, *b*_*wf*_ = 0.245 (red stars).

We have chosen the parameters in model (1) such that the [*FPR*1]/[*BLT*1](5) ratio matches the observed fMLP/LTB4 data (Boribong et al., 2019). We are interested in determining how this balance can be broken and which interactions are responsible for the bimodal switch between equal [*FPR*1] and [*BLT*1] values and dominant [*FPR*1] values. The results presented at *t* = 5 h are transient results. At equilibrium, the [*FPR*1]/[*BLT*1] ratio is 1 for LPS < 15 ng/mL and as large as 10^7^ for LPS = 100 ng/mL. This indicates that all [*BLT*1] molecules have been down regulated, and only [*FPR*1] molecules remain on the surface of neutrophils. This is due to the large non-LPS activation rate of [*GRK*2] protein, *c*_*w*_ = 15. If we either increase the *c*_*w*_ value to *c*_*w*_ = 28 or decrease it to *c*_*w*_ = 5, we maintain the [*FRP*1]/[*BLT*1] ratio 5 h after super-low-dose (1 ng/mL) and high-dose (100 ng/mL), [*FPR*1]/[*BLT*1](5), if we simultaneously decrease the inhibition rate of [*GRK*5] to 5 × 10^−4^ or increase it to 0.25, respectively (see Figure 6). The range of LPS initial conditions that lead to identical [*FPR*1] and [*BLT*1] distribution decrease as *c*_*w*_ decreases, with the [*FPR*1]/[*BLT*1](5) = 1 prediction being lost for *c*_*w*_ = 5 (see Figure 6, red curves). The results are insensitive to the LPS decay rate, or to [*FPR*1] and [*BLT*1] values where phosphorylation and dephosphorylation levels are half-maximal (not shown). These results suggests that the bimodal switch between the [*FPR*1]/[*BLT*1] levels is due to mutual inhibition of GRK2-GRK5 kinases. To confirm this, we removed the inhibition factors, by replacing $\left(c_{w}+a_{w}\left[LPS\right]\right)/\left(b_{fw}+{\left[GRK5\right]}^{n}\right)$ with (*c*_*w*_ + *a*_*w*_[*LPS*])/*b*_*fw*_ and (*c*_*f*_ + *a*_*f*_[*LPS*])/(*b*_*wf*_ + [*GRK*2]) with (*c*_*f*_ + *a*_*f*_[*LPS*])/*b*_*wf*_. When the mutual inhibition is removed, the equilibrium [*FPR*1]/[*BLT*1] levels are constant, and equal to 1,500, regardless of the size of LPS stimulus.

**Figure 7:**
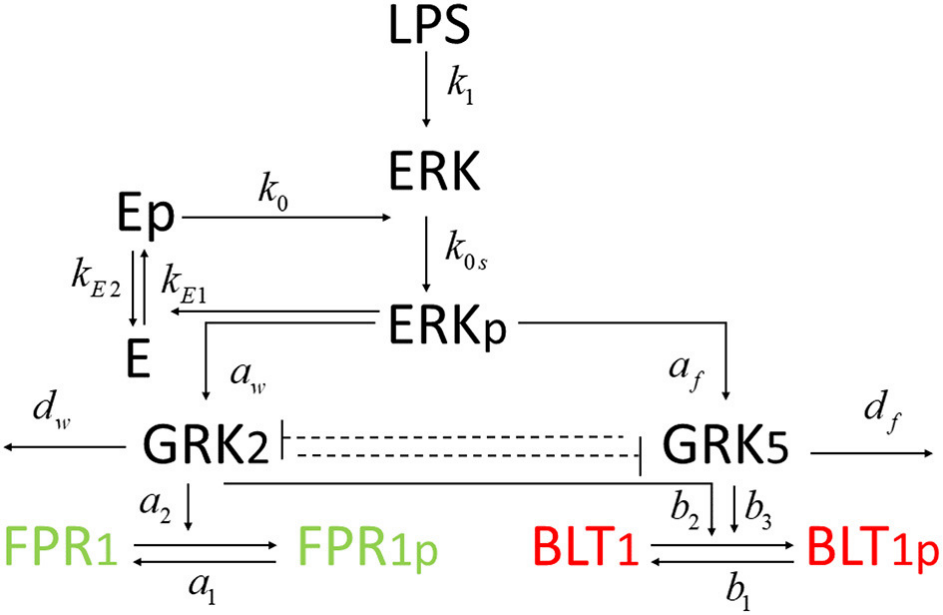
Diagram for model (4).

### 3.3. Molecular Mechanisms of Cell Oscillatory Migration

**Figure 8:**
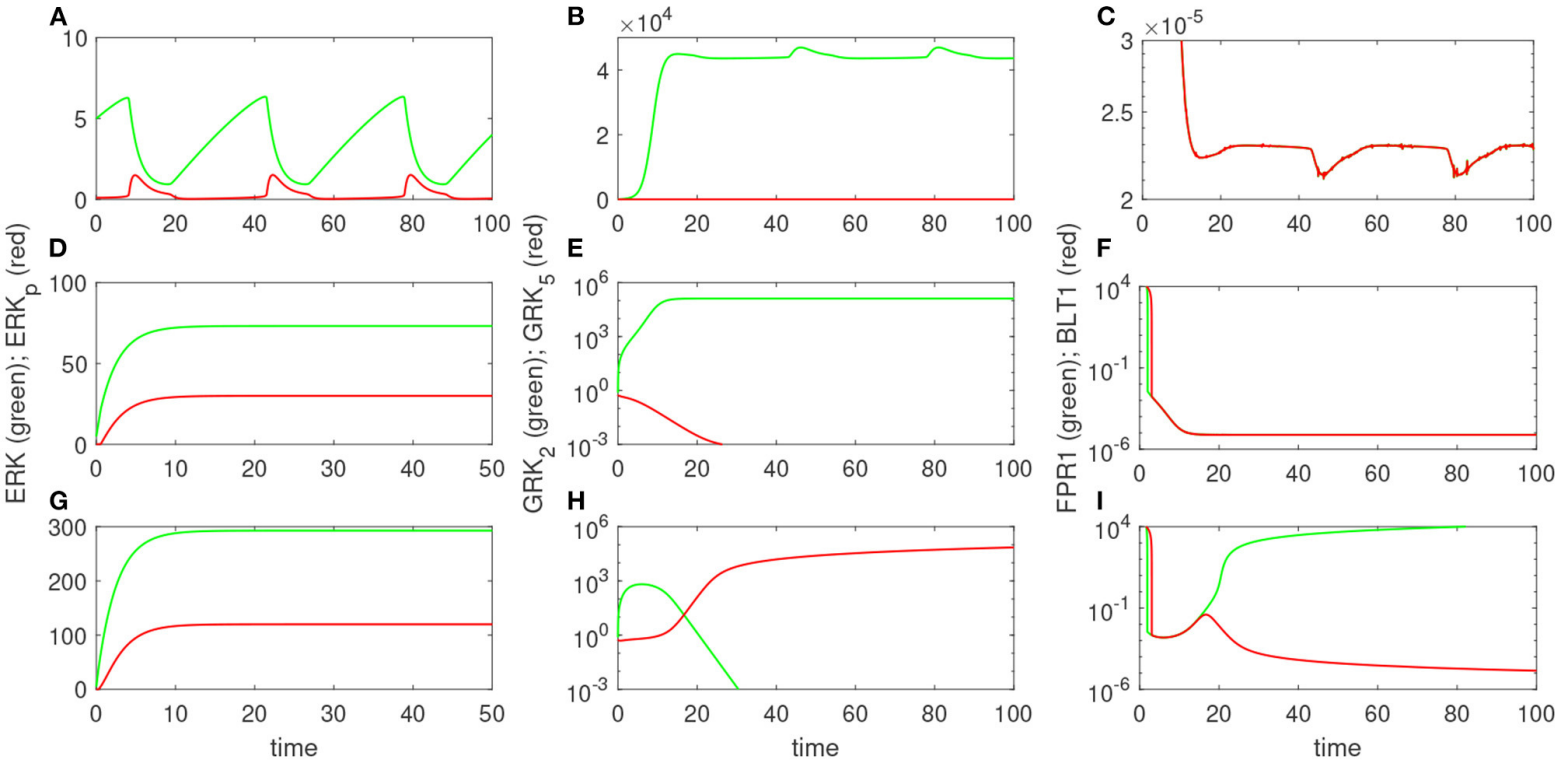
Theoretical results: Dynamics of [*ERK*] and [*ERKp*] **(A,D,G)**; [*GRK*2] and [*GRK*5] **(B,E,H)**; and [*FPR*1] and [*BLT*1] **(C,F,I)**, as given by model (4) for [*LPS*](0) = 1 ng/mL **(A–C)**; [*LPS*](0) = 100 ng/mL **(D–F)**; and [*LPS*](0) = 400 ng/mL **(G–I)**.

Experimental results reported that neutrophils treated overnight with LPS may loose their ability to move up the chemoattractant gradient, become disoriented, and display oscillatory behavior (Boribong et al., 2019). Moreover, the highest number of cells to display such oscillatory behavior occurs following LPS exposure with low dose of 1 mg/ml (see Figure 3B) (Boribong et al., 2019). To determine the molecular mechanisms responsible for the oscillations, we extended the bistable system (1), by coupling it with an activator-inhibitor oscillatory model for the dynamics of non-phosphorylated and phosphorylated extracellular signal-regulated kinases, [*ERK*] and [*ERK*_*p*_], and two auxiliary enzymes, [*E*] and [*E*_*p*_] based on diagram (7), model (4) and parameter values/initial conditions in Tables 1, 2. Moreover LPS is fixed at its initial condition. Under the chosen parameters, we obtain long-term oscillatory movement for all populations, for constant super-low-challenge LPS (1 ng/mL), as predicted by the data (Boribong et al., 2019) (see Figures 8A–C). Interestingly, the oscillatory behavior is maintained for a short LPS range, *(0.5-1.1)* ng/mL; and corresponds to equal distribution of [*FPR*1] and [*BLT*1] receptors. If we either lower the constant LPS challenge to < 0.4ng/mL or increase it to 1.2 − 375ng/mL, we obtain equal density of [*FPR*1] and [*BLT*1] receptors, but no oscillations (see Figures 8D–F). If the LPS constant challenge is increased further, to > 376ng/mL, [*FPR*1] receptors dominate the outcome (see Figures 8G–I). While the switch between low and high [*FPR*1]/[*BLT*1] ratio observed in the constant high dose LPS challenge is due to mutual inhibitions of [*GRK*2] and [*GRK*5] kinases, as observed in model (1), the oscillatory dynamics are due to the oscillatory dynamics of the [*ERK*] and [*ERKp*] kinases. This oscillatory behavior can be broken by either increasing or decreasing the constant LPS challenge. Such information can inform interventions, as dysoriented neutrophil movement is not desirable and has been shown to have negative effects during pathogenic infections. Dysregulated neutrophil response to infection can lead to sepsis and end-organ failure and is a leading cause of death worldwide (Reddy and Standiford, 2010; Shen et al., 2017).

## 4. Discussion

In this study, we developed compartmental mathematical models of molecular interactions that govern neutrophil migratory patterns when exposed to competing chemoattractants and challenged with external stimuli. When the models were restricted to the interactions between the chemoattractants' receptors, their kinases, and LPS, we predicted a bistable switch between two states: one in which the densities of the two chemoattractant receptors, FPR1 and BLT1, are equal and one in which the receptors for the primary chemoattractant, FPR1, dominate. We hypothesized that the two states correspond to two states observed experimentally: equal migration toward the primary and intermediary chemoattractants, fMLP and LTB4, and predominant migration toward the primary chemoattractant, fMLP (Boribong et al., 2019). The experimental data connected the differential migratory outcomes with the magnitude of the external LPS challenge, with super-low 1 ng/mL LPS leading to equal migration toward both chemoattractants and high 100 ng/mL LPS leading to ten times higher migration toward fMLP, 5 h after challenge. In the mathematical model, the external signal corresponds to the initial condition for variable LPS. Our model was calibrated to match the experimental data 5 h after stimuli, with [*FPR*1]/[*BLT*1](5) = 1 for [*LPS*](0) = 1 and [*FPR*1]/[*BLT*1](5) = 10 for [*LPS*](0) = 100. Furthermore, 5 h after LPS challenge, we obtain equal levels of FPR1 and BLT1 receptors for all initial conditions [*LPS*](0) < 3.7 ng/mL and increasingly more FPR1 than BLT1 receptors when the initial condition for LPS increases, with ten times more FPR1 than BLT1 receptors when [*LPS*](0) = 100 ng/mL. When we run the model to equilibrium, however, we obtain equal FPR1 and BLT1 receptors for an even larger range of LPS initial conditions, < 15 ng/ml; and dominant FPR1 levels for LPS> 16 ng/mL. This implies that not just the challenge dose, but the duration of the experiment may influence the quantitative outcomes.

We investigated the molecular interactions that are responsible for the bistable outcomes and found that when the mutual inhibition of GRK2 and GRK5 kinases is either removed, or the balance is broken, the model is no longer bistable. Instead, when run to equilibrium, it settles into a state where FPR1 receptors dominate the outcome, indicative of predominant migration toward the primary chemoattractant.

When the models were expanded to add the interaction with the ERK signaling pathways under constant LPS challenge, we obtained a third dynamical state, where we have equal FPR1/BLT1=1, but the equilibrium is lost, and the FPR1 and BLT1 receptors oscillate between two values. We assume this to be indicative of neutrophil oscillation which, in the experimental setup, is equivalent to cells changing direction (Boribong et al., 2019). We investigated how changes in the constant LPS dose affect the outcomes and found that, at equilibrium, FPR1 and BLT1 receptors are equal and non-oscillating for both super-super-low (< 0.5 ng/mL) and high dose (1.1, 375 ng/mL) LPS; are equal and oscillating for super-low dose (0.5–1.1 ng/mL) LPS; and FPR1 outnumbers BLT1 for super-high dose (> 376 ng/mL) LPS. The non-asymptotic dynamics are due to the oscillatory behavior of phosphorylated and non-phosphorylated ERK molecules, who undergo an auto-catalytic interaction with two undefined enzymes. We have modeled the interaction using an activation-inhibition motif, with similar dynamics being obtained if the oscillations are induced by a substrate depletion motif (Tyson et al., 2003). Further work is needed to determine the nature of enzyme or their regulation. We are currently working to validate the model by retrieving neutrophils from our microfluidic device post-migration and quantifying FPR1, BLT1, GRK2, and GRK 5 levels by Droplet Digital^*TM*^ PCR (ddPCR^*TM*^).

**Figure 9:**
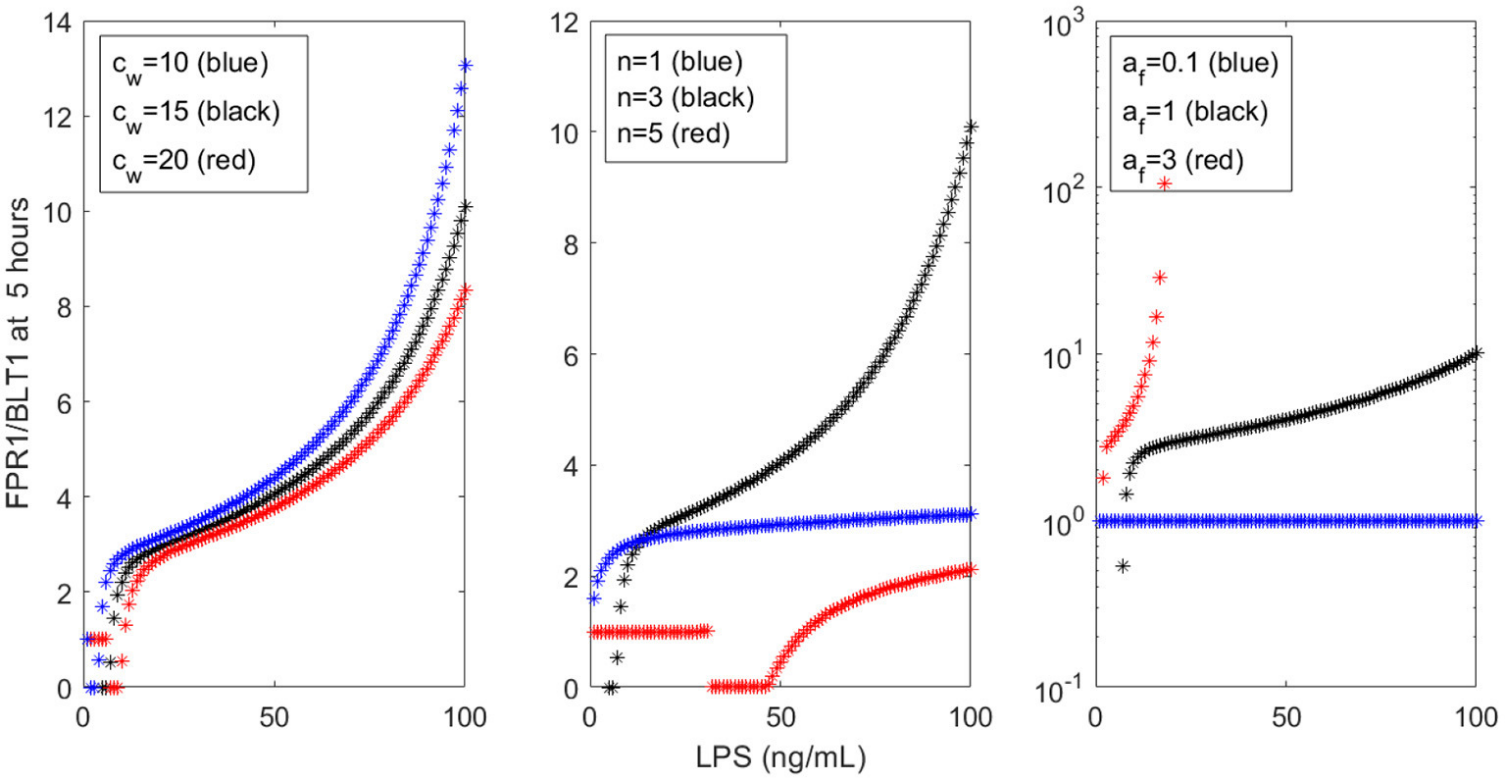
FPR1/BLT1 at time *t* = 5 h, as given by model (1), for **(Left)** *c*_*w*_ = 10 (blue stars), *c*_*w*_ = 15 (black stars), *c*_*w*_ = 20 (red stars); **(Middle)** *n* = 1 (blue stars), *n* = 3 (black stars), *n* = 5 (red stars); and **(Right)** *a*_*f*_ = 0.1 (blue stars), *a*_*f*_ = 1 (black stars), *a*_*f*_ = 3 (red stars).

Our models are limited by the presence of many unknown parameters. While we strived to match the empirical data, the results are mostly qualitative. Similar results can be obtained with many different parameters sets. For example, *c*_*w*_ only slightly influences the FPR1/BLT1 ratio at *t* = 5 h (see Figure 9, left panel), while the cooperativity coefficient *n* and the LPS-dependent GRK5 production *a*_*f*_ have drastic effects on the size of the ration (see Figure 9, middle and right panels). The unchanging factors, however, are the motifs of bistability, which are induced by the dual inhibition of the G-protein kinases; the oscillatory motifs, which are induced by the oscillatory ERK dynamics; and the influence of external stimuli on outcomes.

In conclusion, we developed mathematical models for the molecular interactions responsible for neutrophils migratory phenotypes, calibrated them against empirical data, and used their dynamics to determine the external stimuli ranges that account for neutrophils migration toward a pro-inflammatory chemoattractant, a pro-tolerant chemoattractant, or oscillatory dynamics indicative of dysorientation and loss of function. Understanding the relationship between neutrophils' dynamics and the mechanisms responsible for their movement is important for preventing and predicting immune disorders. Activation markers in neutrophils are potential biomarkers for the diagnosis and prognosis of sepsis. Septic patients can be screened for neutrophil markers, such as GRK 2/5, FPR1, and BLT1, and this predictive model can guide patient treatment. In the future, we plan to expand this model to include more complex signaling from the microenvironment and aim to predict not only dysfunctional migration in neutrophils, but even the probability of cells accumulating in specific organs, such as the lung or kidney. We can use these predictive models to define optimal patient treatment and to identify immunotherapeutic targets (i.e., small molecule inhibition, microRNAs, gene therapy) to promote directional neutrophil migration.

## Data Availability Statement

The original contributions presented in the study are included in the article/supplementary material, further inquiries can be directed to the corresponding author/s.

## Author Contributions

SC, SK, and BB wrote the code. SC wrote the manuscript. All authors reviewed and revised the manuscript and conceived the study.

## Conflict of Interest

The authors declare that the research was conducted in the absence of any commercial or financial relationships that could be construed as a potential conflict of interest.
